# Impact of interhospital transfer vs. direct admission on acute ischemic stroke patients: A subset analysis of the COMPLETE registry

**DOI:** 10.3389/fneur.2022.896165

**Published:** 2022-08-09

**Authors:** Ameer E. Hassan, Osama O. Zaidat, Ashish Nanda, Benjamin Atchie, Keith Woodward, Arnd Doerfler, Alejandro Tomasello, Johanna T. Fifi

**Affiliations:** ^1^Valley Baptist Medical Center, Neuroscience Department, University of Texas Rio Grande Valley, Harlingen, TX, United States; ^2^Endovascular Neurology and Neuroscience, Mercy Health St. Vincent Medical Center, Toledo, OH, United States; ^3^SSM St. Clare Healthcare, Fenton, MO, United States; ^4^RIA Neurovascular, Englewood, CO, United States; ^5^Fort Sanders Regional Medical Center, Knoxville, TN, United States; ^6^Department of Neuroradiology, Universitätsklinikum Erlangen, Erlangen, Germany; ^7^Neurorradiologia Intervencionista, Hospital Universitari Vall d'Hebron, Barcelona, Spain; ^8^Icahn School of Medicine at Mount Sinai, New York, NY, United States

**Keywords:** acute ischemic stroke, stroke systems of care, aspiration thrombectomy, interhospital transfer, large vessel occlusion (LVO)

## Abstract

**Background:**

Efficacy of thrombectomy treatment in acute ischemic stroke large vessel occlusion (AIS-LVO) patients is time dependent. Direct admission to thrombectomy centers (vs. interhospital transfer) may reduce time to treatment and improve outcomes. In this subset analysis of the COMPLETE registry, we compared outcomes between direct to thrombectomy center (Direct) vs. transfer from another hospital to thrombectomy center (Transfer) in AIS-LVO patients treated with aspiration thrombectomy.

**Methods:**

COMPLETE was a prospective, international registry that enrolled patients from July 2018 to October 2019, with a 90-day follow-up period that was completed in January 2020. Imaging findings and safety events were adjudicated by core lab and independent medical reviewers, respectively. Pre-defined primary endpoints included post-procedure angiographic revascularization (mTICI ≥2b), 90-day functional outcome (mRS 0–2), and 90-day all-cause mortality. Planned collections of procedural time metrics and outcomes were used in the present *post-hoc* analysis to compare outcomes between transfer and direct patient cohorts.

**Results:**

Of 650 patients enrolled, 343 were transfer [52.8% female; mean (SD) age, 68.2 (13.9) years], and 307 were direct [55.4% female; 68.5 (14.5) years] admit. Median onset-to-puncture time took longer in the transfer vs. direct cohort (5.65 vs. 3.18 h: 2.33 h difference, respectively; *p* < 0.001). There was no significant difference in successful revascularization rate, mTICI ≥2b (88.3 and 87.3%), sICH at 24 h (3.8 and 3.9%), median length of hospital stay (7 and 6 days), and 90-day mortality (16.9 and 14.0%) between transfer vs. direct patients, respectively. However, achieving 90-day functional independence was less likely in transfer compared with direct patients (mRS 0–2 was 50.3 vs. 61.7%, *p* = 0.0056).

**Conclusions:**

In the COMPLETE registry, direct to thrombectomy center was associated with significantly shorter onset-to-puncture times, and higher rates of good clinical outcome across different geographies. Additional research should focus on AIS-LVO detection to facilitate direct routing of patients to appropriate treatment centers.

**Clinical trial registration:**

https://clinicaltrials.gov (Unique identifier: NCT03464565).

## Introduction

Endovascular mechanical thrombectomy (MT) represents the current standard of care for acute ischemic stroke secondary to large vessel occlusion (AIS-LVO) (1, 2).

Treatment outcomes for thrombolysis and thrombectomy are time sensitive (1). A recent meta-analysis showed that the benefits of endovascular thrombectomy are most pronounced in patients treated within 2 h of symptom onset, with each additional 1-h delay associated with lower functional independence (mRS 0–2) (3).

Current systems of care for patients with suspected LVO involve pre-hospital triage which routes patients either directly to a stroke center capable of mechanical thrombectomy, or initially to a nearer hospital without endovascular capability, but which may still offer intravenous tissue plasminogen activator (IV-tPA). It is therefore important to understand whether centers offering only IV-tPA should be bypassed for direct admission to comprehensive stroke centers offering mechanical thrombectomy.

In many circumstances, direct admission to thrombectomy centers (as compared with interhospital transfer) may improve clinical and functional outcomes (4–6). In STRATIS, a prospective registry, patients directly admitted to an endovascular-capable center had significantly reduced treatment delays and a better chance of achieving functional independence, as compared to transfer patients (7). Furthermore, IV-tPA did not significantly affect outcomes in this cohort. Hypothetical bypass modeling suggested that although IV-tPA would be delayed by an average of 12.0 min, endovascular treatment would be delivered 91.0 min sooner, had patients been directly routed to endovascular-capable centers (7).

By contrast, RACECAT, a randomized controlled trial conducted in Catalonia region of Spain, reported that both direct and transfer pre-hospital protocols yielded similar 90-day mRS outcomes in both patient cohorts (8).

Many U.S. and European geographies however, lack unified stroke transfer protocols, and patient transport is subject to regional emergency medical services or triage policy (9, 10). This variability contrasts with the pre-hospital triage efficiency of RACECAT, which was run within a well-integrated public health network (8).

In this subset analysis, we analyzed outcomes from patients enrolled in the COMPLETE registry who were either directly admitted, or secondarily transferred, to a thrombectomy capable center. This trial included 42 sites from North America (29) and Europe (13), comprising six countries, 12 European cities, and 17 U.S. states across major metropolitan and rural catchments (1, 11, 12).

This study was designed to assess systems of care across different geographies, and included both anterior and posterior LVO patients with no restrictions on stroke onset time.

## Materials and methods

### Study design and participants

COMPLETE was a global, prospective, multicenter, single-arm, observational registry assessing the performance and safety of the Penumbra System in a patient population with AIS-LVO (ClinicalTrials.gov Identifier: NCT03464565). The data supporting the findings of this study are available from the corresponding author upon reasonable request.

Inclusion criteria were: (1) patient age ≥18 years, (2) pre-stroke mRS 0–1, (3) Patient experiencing AIS secondary to intracranial LVO who are eligible for MT using the Penumbra System, (4) Planned frontline treatment with Penumbra System, and (5) Signed informed consent per Institution Review Board/Ethics Committee.

Exclusion criteria were: (1) Any comorbid disease or condition expected to compromise survival or ability to complete follow-up assessments through 90 days, and (2) Currently participating in an investigational (drug, device, etc.) clinical trial that will confound registry endpoints. Patients in observational, natural history, and/or epidemiological studies not involving intervention are eligible.

Participating hospitals maintained a screening and enrollment log of all AIS-LVO patients admitted into the hospital who were eligible for MT. Reason(s) for exclusion were recorded. Patients were considered enrolled once informed consent was obtained per Institutional Review Board/Ethics Committee and Penumbra System had been inserted into the body. Additional details on the COMPLETE Registry are reported separately (13).

Patients were either admitted directly to an enrolling hospital (direct) or transferred from an outside facility to the enrolling hospital (transfer). The decision for direct or transfer admission to a thrombectomy center was made by the stroke response procedures followed by the emergency services for each enrolling hospital and not dictated by the COMPLETE registry's protocol.

The choice of frontline treatment using direct aspiration only or aspiration combined with a 3D Revascularization Device was made by the treating physician. All procedures were conducted in accordance with routine care at each participating hospital and the Instructions for Use for each device. Available devices included: the Penumbra MAX, ACE, and JET Reperfusion Catheters; the 3D Revascularization Device; and the Penumbra Pump MAX, and ENGINE aspiration sources.

### Registry outcomes

The primary efficacy endpoints were: angiographic revascularization of the occluded target vessel at immediate post-procedure as defined by a modified thrombolysis in cerebral infarction (mTICI) score of 2b or higher, and, good functional subject outcome at 90 days post-procedure as defined by a modified Rankin Score (mRS) 0–2. The primary safety endpoint was all-cause mortality at 90 days.

Secondary endpoints included: Incidence of device- and procedure-related Serious Adverse Events (SAEs) at ≤24 h, occurrence of embolization in previously uninvolved or new territories (ENT) as seen on the final control angiogram at the end of procedure, occurrence of symptomatic intracranial hemorrhages (sICH) at 24 h, vessel perforation, vessel dissection, length of hospital stay, and time metrics including onset to puncture, door to puncture and puncture to revascularization median times.

### Study committees

An independent imaging core lab reviewed pseudonymized angiography for mTICI scores by pass, embolization of new territory, pre-procedure CT for ASPECTS, CTA for clot location, and 24-h CT to assess hemorrhagic transformation using European Cooperative Acute Stroke Study (ECASS) classification.

Independent medical reviewers (IMRs) reviewed and adjudicated study endpoint events, including device related SAEs, neurovascular procedure related SAEs, sICH within 24 h, neurological deterioration events, and any deaths that occurred throughout the registry. Neurological deterioration was defined as a ≥4 point worsening of the National Institutes of Health stroke scale (NIHSS) score from baseline and sICH was defined as 24-h evidence of an ECASS defined ICH associated with a ≥4 point worsening of the NIHSS score from baseline.

### Statistical analysis

Data was summarized using descriptive statistics. This included the number of observations, mean, standard deviation, median, and interquartile range (IQR) for continuous variables, and counts and percentages for discrete variables. Comparisons between direct and transfer patient groups were conducted using the *t*-test or Wilcoxon Rank-Sum Test for continuous variables and Fisher's Exact Test for categorical variables. All confidence intervals presented are Clopper-Pearson two-sided intervals. All statistical tests were performed using SAS version 9.4 (SAS Institute, Cary, NC) with α = 0.05.

Multivariable logistic regression models with an interaction term for onset-to-puncture time by cohort, were used to examine the variables associated with 90-day mRS. Candidate clinical predictors included cohort (direct vs. transfer), onset-to-puncture time (by 30 min intervals), age (by 10 year intervals), revascularization success (TICI2b-3 post-procedure), occlusion location, tPA (administered vs. not), baseline NIHSS (by 5 point intervals), hypertension (present vs. not), geographic region (US vs. Europe), baseline ASPECTS (<8 vs. ≥8). This model included 250 patients (direct *n* = 129, transfer *n* = 121) with witnessed stroke events and successful revascularization. Patients with onset-to-puncture times >600 min were statistical outliers and excluded.

## Results

From July 2018 to October 2019, the COMPLETE registry screened 1,501 patients and enrolled 650 patients across 42 sites, with 90 days follow up completed in January 2020. Of the 851 screen failures not enrolled in the registry, 58 met entry criteria but declined to participate, 539 did not meet eligibility entry criteria, 78 met entry criteria and consented but did not have any component of the study devices introduced into the body, and 176 were not enrolled for other reasons. The main reasons for exclusion were mRS >1 and planned frontline treatment with non-Penumbra System devices. Study completion rate through the final follow-up assessment was high (94.6%, 615/650), with an attrition rate of 5.4% (35/650). Of the 35 patients that did not complete the study, five withdrew consent, two were withdrawn by a study investigator, 25 were lost to follow-up, and three did not complete for other reasons.

Of 650 enrolled patients, 307 were treated directly at an endovascular-capable hospital and 343 were transferred (Table 1). Baseline characteristics were similar between the direct and transfer patient cohorts, except for hypertension, which was more prevalent in the transfer cohort (*p* < 0.01), and median ASPECTS, which was lower in the transfer cohort (*p* < 0.001; Table 1). Furthermore, the proportion of patients with direct admissions was lower in the US (42.1%) than in Europe (42.1% vs. 59.2%, *p* < 0.001).

**Table 1:** Baseline characteristics.

**Baseline characteristics**	**Direct (*N* = 307)**	**Transferred (*N* = 343)**	***p*-Value**
**Age, mean (SD)**	68.5 (14.5)	68.2 (13.9)	0.7808
**Female, % (** ***n*** **/** ***N*** **)**	55.4% (170/307)	52.8% (181/343)	0.5288
**Symptom onset determination, % (** ***n*** **/** ***N*** **)**
Witnessed	54.7% (168/307)	49.6% (170/343)	0.2084
Wake up stroke	9.1% (28/307)	10.8% (37/343)	0.5144
Unwitnessed: time last seen well	36.2% (111/307)	39.1% (134/343)	0.4662
**Geographic location, % (** ***n*** **/** ***N*** **)**
US	62.2% (191/307)	76.7% (262/343)	<0.0001
EU	37.8% (116/307)	23.3% (80/343)	<0.0001
**Medical history, % (** ***n*** **/** ***N*** **)**
Ischemic stroke	14.0% (43/307)	15.7% (54/343)	0.5820
Hemorrhagic stroke	0.3% (1/307)	0.3% (1/343)	1.0000
Cardiovascular disease	51.8% (159/307)	49.9% (171/343)	0.6379
Diabetes	23.5% (72/307)	23.9% (82/343)	0.9265
Renal failure	6.8% (21/307)	4.4% (15/343)	0.1748
Hypertension	67.1% (206/307)	76.4% (262/343)	0.0088
Hyperlipidemia	41.4% (127/307)	43.1% (148/343)	0.6910
**IV tPA given, % (** ***n*** **/** ***N*** **)**	47.6% (146/307)	50.7% (174/343)	0.4328
**NIHSS, median [IQR]**	14.0 [9.0, 20.0]	15.0 [9.0, 20.0]	0.7183
**ASPECTS, median [IQR]** ^a^	8.0 [7.0, 10.0]	8.0 [7.0, 9.0]	<0.0001
**pc-ASPECTS, median [IQR]** ^b^	9.0 [8.0, 10.0]	9.0 [8.0, 10.0]	0.9579
**Occlusion location, % (** ***n*** **/** ***N*** **)**
ICA	4.2% (13/307)	5.0% (17/343)	0.7111
ICA-T	12.4% (38/307)	13.1% (45/343)	0.8145
M1	55.0% (169/307)	55.4% (190/343)	0.9372
M2	18.6% (57/307)	16.3% (56/343)	0.4694
M3–M4	1.6% (5/307)	1.5% (5/343)	1.0000
A1–A2	1.0% (3/307)	0.3% (1/343)	0.3484
Basilar	4.9% (15/307)	5.2% (18/343)	0.8600
Vertebral	0.3% (1/307)	0.0% (0/343)	0.4723
PCA	2.0% (6/307)	2.9% (10/343)	0.4602

^a^N_direct_ = 283, *N*_transfer_ = 314.

^b^*N*_direct_ = 22, *N*_transfer_ = 27.

### Procedural characteristics

**Figure 1:**
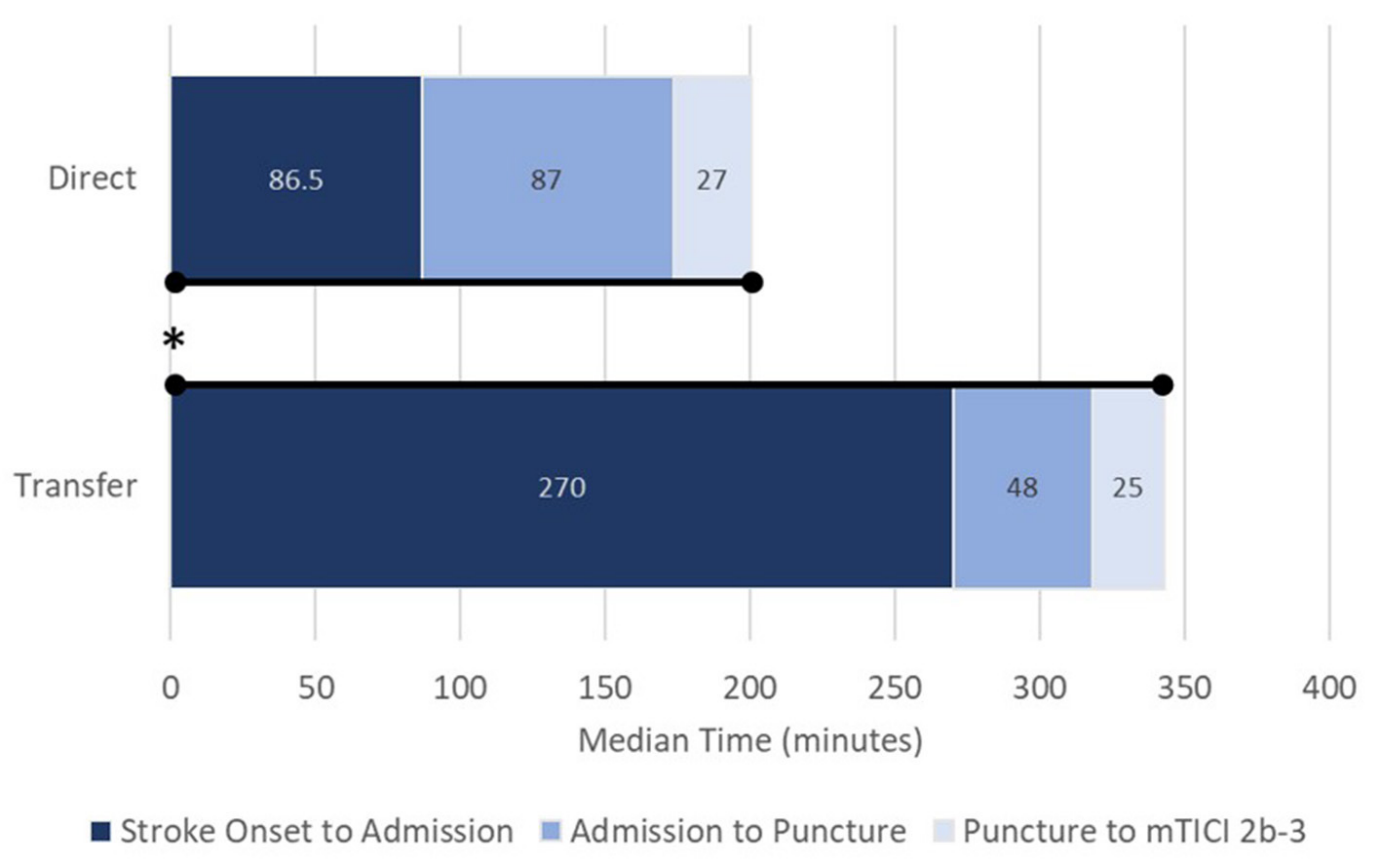
Process times from stroke onset to revascularization (mTICI 2b-3) for direct (upper) and transfer patients (lower). Data presented as medians. mTICI, modified thrombolysis in cerebral infarction; *p < 0.0001 for overall onset to revascularization. Stroke-onset-to-admission, *p* < 0.0001; admission-to-puncture, *p* < 0.0001; puncture-to-revascularization, not significant.

Patients treated directly at a comprehensive stroke center had shorter onset-to-admission times (86.5 vs. 270.0 min), onset-to-puncture times (191.0 vs. 339.0 min), and onset to mTICI 2b-3 else final angiogram times (242.0 vs. 373.0 min) as compared with patients transferred secondarily (*p* < 0.0001 for all comparisons; Table 2; Figure 1). There was no significant difference in procedure time (puncture to mTICI 2b-3) between direct and transfer cohorts (Table 2); however, admission-to-puncture times were longer in the direct cohort, compared to transfer patients (*p* < 0.0001).

**Table 2:** Procedural characteristics.

**Procedural characteristics**	**Direct (*N* = 307)**	**Transferred (*N* = 343)**	***p*-Value**
**First line treatment**
Direct aspiration	65.8% (202/307)	60.3% (207/343)	0.1669
Penumbra catheter with 3D	32.2% (99/307)	37.9% (130/343)	0.1394
**Time metrics, median [IQR]**
Onset-to-Hospital Admission (min)	86.5 [45.0, 241.0]	270.0 [180.0, 474.0]	<0.0001
Admission-to-puncture (min)	87.0 [66.0, 115.0]	48.0 [32.0, 79.5]	<0.0001
Onset-to-puncture (min)	191.0 [138.0, 388.0]	339.0 [248.0, 547.0]	<0.0001
Puncture-to-mTICI 2b-3 (min)	27.0 [15.0, 41.0]	25.0 [16.0, 40.0]	0.6937
Onset-to-mTICI 2b-3 else final angiogram (min)	242.0 [163.0, 433.0]	373.0 [269.0, 586.0]	<0.0001

### Outcomes

**Figure 2:**
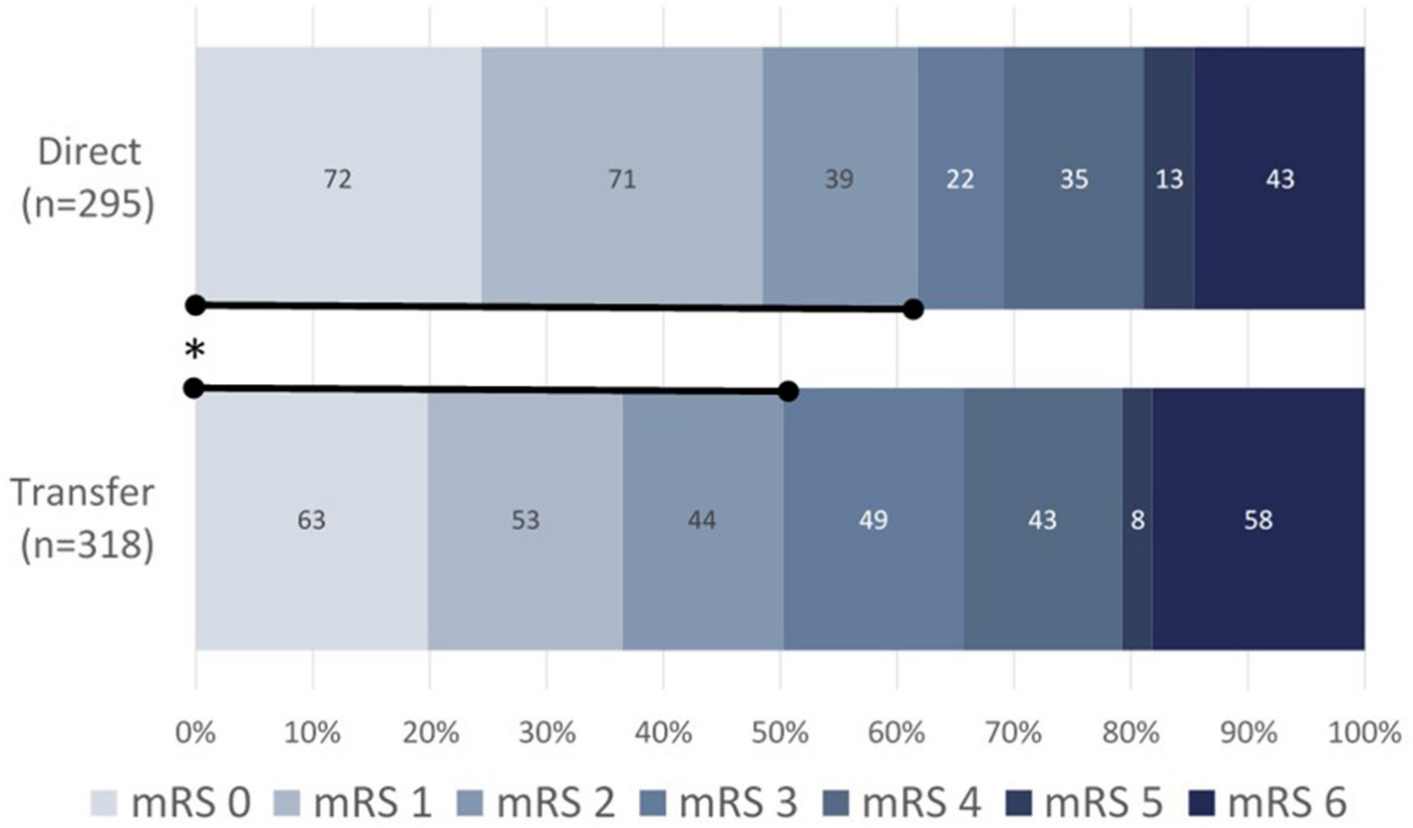
90-day functional outcomes as measured by mRS for direct (upper, *n* = 295) vs. transfer (lower, *n* = 318) patients. Data presented as a percentage of direct admissions vs. interhospital transfer patients. mRS, modified Rankin Scale; *p < 0.0056 for mRS 0–2 direct (61.7%) vs. transfer (50.3%).

Out of 650 enrolled subjects, 613 had a 90-day follow-up assessment. Functional independence at 90 days (mRS 0–2) was significantly higher in the direct vs. transfer group [61.7% vs. 50.3% (+11.4%); *p* = 0.0056; Table 3; Figure 2]. There were no significant differences in post-procedural revascularization rates, nor in all-cause mortality at 90 days between direct and transfer cohorts.

**Table 3:** Endpoints.

**Endpoints**	**Direct (*N* = 307)**	**Transferred (*N* = 343)**	***p*-Value**
mTICI 2b-3 post-procedure	87.3% (268/307)	88.3% (303/343)	0.7192
mRS 0–2 at 90 days	61.7% (182/295)	50.3% (160/318)	0.0056
All-cause mortality at 90 days	14.0% (43/307)	16.9% (58/343)	0.3302
**Secondary safety endpoints, % (** ***n*** **/** ***N*** **) (95% CI)**
Device related SAE ≤24 h	0.3% (1/307)	0.9% (3/343)	0.6262
Procedure related SAE ≤24 h	5.5% (17/307)	6.1% (21/343)	0.8673
Embolization in previously uninvolved or new territories (ENT)	2.9% (9/307)	2.6% (9/343)	0.8162
Symptomatic intracranial hemorrhage (sICH) ≤24 h	3.9% (12/307)	3.8% (13/343)	1.0000
Vessel dissection	1.0% (3/307)	0.9% (3/343)	1.0000
Vessel perforation	0.7% (2/307)	0.0% (0/343)	0.2227
Length of hospital stay in days, median [IQR]	6.0 [3.0, 11.0]	7.0 [4.0, 11.0]	0.1091

Direct and transfer cohorts did not differ in rates of device- or procedure- related SAE within 24 h, nor in rates of procedural ENT, vessel dissection, or vessel perforation, and 24 h sICH (Table 3). The length of hospital stay was also similar between direct and transfer patients (6.0 vs. 7.0 days).

**Figure 3:**
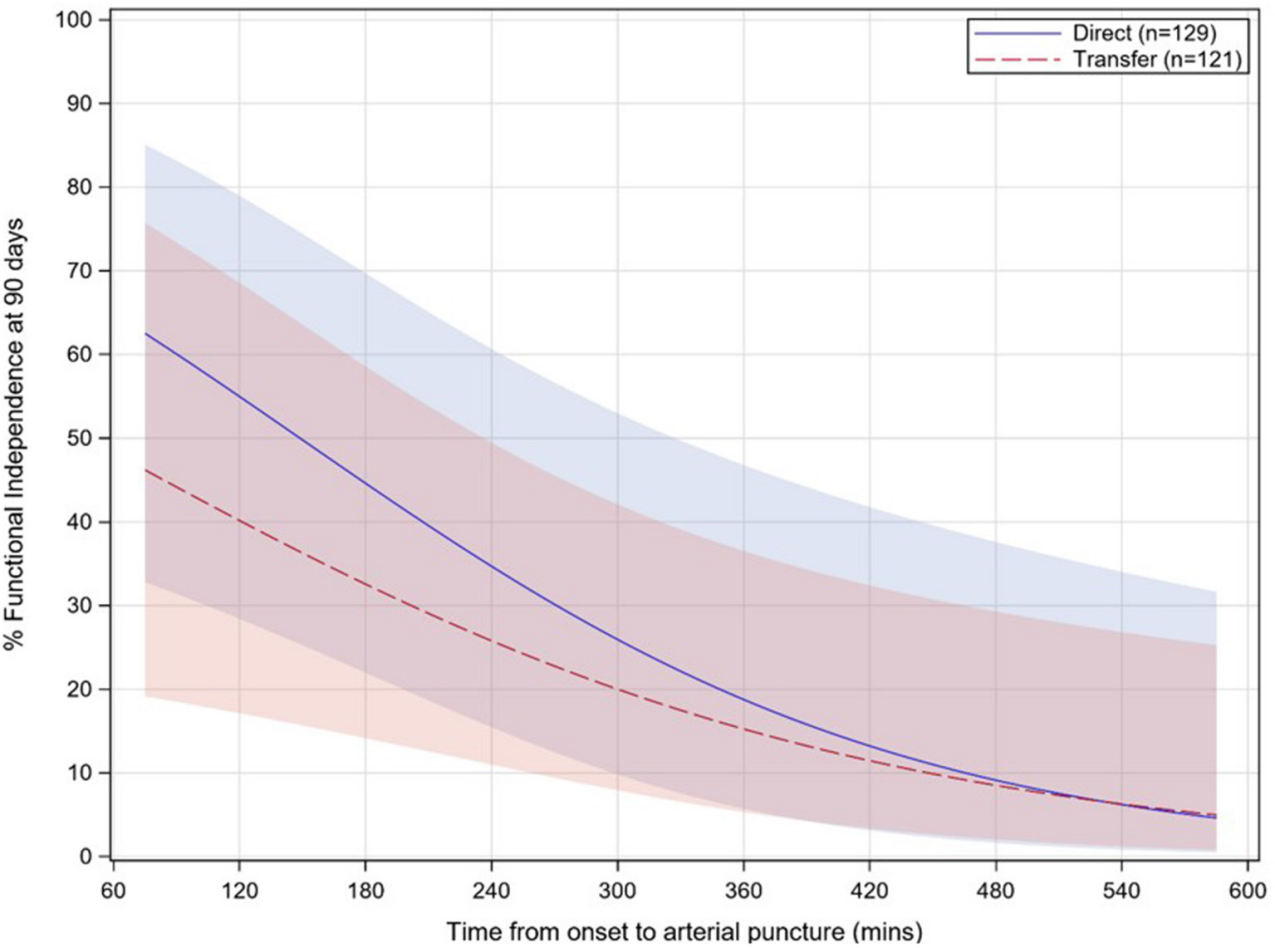
Relationship between functional independence rates (90 day mRS 0–2) and onset-to-puncture time for direct (solid, blue) vs. transfer (dashed, red) patients. Onset-to-puncture times >1.5 times the interquartile range are not displayed. Shaded areas for each group represent 95% confidence intervals. mRS, modified Rankin Scale; *p* = 0.6612 for interaction.

In the multivariable analysis, independent predictors of good functional outcome (mRS 0–2) included lower baseline NIHSS, baseline ASPECTS ≥8, IV-tPA administration, lower age, a distal occlusion location (e.g. M2–M4, instead of M1), and faster onset-to-puncture times (Table 4). For onset-to-puncture time, the odds of patients achieving good functional outcome decreased by 0.85 for every 30 min that treatment was delayed (*p* = 0.0180; Table 4). No interaction effect was observed between the direct admit cohort and onset-to-puncture time (*p* = 0.6612; Figure 3). The effect of direct admission on rates of mRS 0–2 did not persist after controlling for time from onset to puncture, nor other baseline and procedural covariates. For patients who were directly admitted, the odds of good functional outcome were higher [adjusted OR 2.17 (95% CI: 0.42, 11.3)] than for transfer patients; however, this effect was not significant (*p* = 0.3584).

**Table 4:** Unadjusted and adjusted logistic regression modeling for odds of achieving good functional outcome (mRS 0–2 at 90 days).

**Variables**	**Unadjusted**		**Adjusted**	
	**Odds ratio (95% CI)**	***p*-Value**	**Odds ratio (95% CI)**	***p*-Value**
Cohort (Direct)	1.59 (1.15, 2.19)	0.0047	2.17 (0.42, 11.3)	0.3584
tPA (Yes)	1.46 (1.06, 2.02)	0.0202	2.10 (1.05, 4.20)	0.0364
NIHSS (per 5 pts)	0.63 (0.55, 0.71)	<0.0001	0.49 (0.37, 0.64)	<0.0001
ASPECTS (≥ 8)	2.28 (1.61, 3.23)	<0.0001	2.07 (1.03, 4.15)	0.0399
Hypertension (Yes)	0.58 (0.40, 0.83)	0.0031	1.37 (0.63, 2.97)	0.4327
Region (EU)	1.04 (0.74, 1.46)	0.8332	1.19 (0.61, 2.34)	0.6070
Time: Onset-To-Puncture (per 30 mins increase)	0.98 (0.97, 1.00)	0.0365	0.85 (0.74, 0.97)	0.0180
Interaction: Onset-To-Puncture vs. Cohort	1.00 (1.00, 1.00)	0.8173	1.00 (0.99, 1.01)	0.6612
Age (per 10 yr increase)	0.66 (0.58, 0.75)	<0.0001	0.44 (0.33, 0.60)	<0.0001
TVL: (ACA) vs. M1 ref.	0.80 (0.11, 5.72)	0.8214	0.21 (0.01, 4.82)	0.3283
TVL: (ICA) vs. M1 ref.	0.62 (0.40, 0.96)	0.0341	0.99 (0.44, 2.21)	0.9755
TVL: (M2-M4) vs. M1 ref.	1.84 (1.18, 2.87)	0.0076	2.83 (1.12, 7.13)	0.0275

ACA, anterior cerebral artery; ASPECTS, Alberta stroke program early CT score; CI, confidence interval; EU, European union; ICA, internal carotid artery; M1 initial segment of the middle cerebral artery; M2-M4, second to fourth segment of the middle cerebral artery; NIHSS, National Institutes of Health stroke scale; tPA, tissue plasminogen activator (Alteplase); TVL, target vessel location.

## Discussion

In the COMPLETE Study, functional independence was improved for AIS-LVO patients directly admitted to an endovascular-capable center, compared with those who were transferred (90-day mRS 0–2: 61.7 vs. 50.3%; Table 3). Furthermore, patients directly admitted to comprehensive stroke centers had faster onset-to-puncture times (191.0 vs. 339.0 min; Table 2), which is acknowledged as a predictor of better clinical outcomes in the American Heart and American Stroke Association guidelines (1).

### Effect of direct vs. transfer on good functional outcome

Results from COMPLETE show that direct admission can significantly benefit functional outcome in AIS-LVO patients treated with frontline aspiration thrombectomy, and mirror two similar prospective registries in which mechanical thrombectomy was performed using stent retrievers (7), or a combination of stent-retriever or contact aspiration firstline (6). The magnitude of this effect was consistent across studies, with ~10% more patients achieving mRS 0–2 at 90 days in the direct vs. transfer cohorts: COMPLETE 61.7 and 50.3%, respectively; STRATIS 60.0 and 52.2% (7); and, ETIS 60.1 and 52.6% (6).

To further understand the benefit of direct admission on good functional outcome (mRS 0–2), a multivariable logistic regression was used to adjust for baseline and procedural characteristics (Table 4). In this analysis, the effect of direct admission was non-significant (*p* = 0.3584), suggesting that the benefits of direct admission occur indirectly, either from variation in tPA administration, baseline NIHSS and ASPECTS, occlusion location, or, based on predictive modeling presented in this study, from the time interval between stroke onset to puncture (Table 4). Furthermore, no significant interaction effect was found between patient cohort and onset-to-puncture time, supporting results that the benefits of direct admission can be attributed to delays in patient treatment (Table 4).

### Effect of direct vs. transfer on the admission-to-puncture interval

In the COMPLETE Study, the admission-to-puncture interval actually took significantly longer in the direct admission patients (87 min) compared with the transfer patients (48 min; *p* < 0.001; Table 3). In the ARTESp study (14), mechanical thrombectomy was initiated significantly faster for transfer patients following arrival at an endovascular-capable center, due to the availability of imaging. Although this did not compensate for the lost time incurred by secondary transport (direct admission was twice as likely to yield a favorable clinical outcome), this is consistent with the admission-to-puncture data from COMPLETE and the recognition that multiple variables can account for the improved clinical outcomes observed in direct admission patients.

### Effect of direct vs. transfer on technical efficacy and safety outcomes

Revascularization success (mTICI 2b-3), 90-day mortality, and rates of sICH, did not differ between direct vs. transfer cohorts in the COMPLETE registry. These results are again consistent with recent studies reporting similar revascularization and safety outcomes regardless of patient transfer status (6, 7).

Our results are further supported by recent meta-analyses reporting no significant differences found between the two transportation paradigms in the rate of sICH, mortality at 3 months, or in successful recanalization (15, 16).

For AIS-LVO patients eligible for mechanical thrombectomy, treatment delays can result in unfavorable clinical outcomes (17). Therefore, triage routing should focus on improving interval times and reducing time imaging and revascularization (18). For example, the interval between symptom onset and reperfusion might be reduced by bringing transfer patients directly to the angiosuite, a possibility supported by data showing intrahospital processing times were nearly twice as quick for transferred patients. Nevertheless, in the COMPLETE registry, patients who were secondarily transferred to an endovascular-capable center experienced longer onset-to-hospital admission times, longer onset-to-puncture times, and longer onset-to-revascularization times. When comparing the symptom onset-to-revascularization intervals between the direct (242.0 min) and transfer (373.0 min) cohorts, patients experienced a delay of 131 min if they were secondarily transported to an endovascular-capable center (Table 2).

This time delay is not surprising and compares to a median onset-to-revascularization delay in transfer patients of 109 min (311.5 vs. 202.0 min) in STRATIS (7), and of 100 min (219.0 vs. 319.0 min) for transferred patients in ETIS (6). When controlling for time from onset-to-treatment, the authors in STRATIS found no difference in outcome between direct and transfer patients, indicating for that registry population, that improved functional outcome was attributed solely to time delays associated with the transfer paradigm (7). Results from the COMPLETE registry corroborate this analysis, with direct admissions significantly associated with both faster onset-to-revascularization times, as well as better functional outcomes.

The results observed in COMPLETE are notable in that the overall effect on functional outcome remained comparable to registries in which transfer patients were treated approximately 1 to 2.5 h earlier than those in COMPLETE (6, 7). In COMPLETE, time from onset-to-treatment for the highest quartile was just over 9 h (547.0 min; Table 2); despite this, a significant benefit was still observed between direct and transfer cohorts. By contrast, controlled trials for late-presenting strokes (19, 20) reported equivalent benefits for both direct admissions and for transfer patients. Nevertheless, the authors note the selective nature of the patient population for penumbral mismatch and likely over-representation of candidates with favorable collaterals (19, 20), emphasizing the need for nuanced, controlled studies of transportation paradigms for AIS-LVO.

### Effects of tPA on direct and transfer

IV-tPA administration was an independent predictor of better functional outcomes for patients overall (Table 4), although there was no significant difference in IV-tPA administration across direct and transfer patients (Table 1).

Froehler et al. (7) remarks that although the ability to begin IV-tPA sooner is often cited as a reason to bring patients to a nearer hospital, direct-to-endovascular bypass may still be beneficial because it accelerates the start of endovascular therapy. For example, when modeling a bypass <20 miles to an endovascular-capable center, tPA is delayed by only 6.9 min, while endovascular treatment to begin 94 min sooner (7).

Nevertheless, the use of bridging thrombolysis when appropriate may alleviate the difference in transport paradigms, potentially by compensating for the negative effects of delayed reperfusion time: Zhao et al. (16) found in a subgroup analysis of that bridging thrombolysis resulted in similar outcomes for direct and transfer cohorts with respect to sICH, 90-day favorable functional outcome, 90-day mortality, and successful recanalization. In practice, however, the authors concur that for patients ineligible for IV thrombolysis, direct transport to the closest endovascular-capable center may be the best option (16).

Our results establish that AIS-LVO patients treated with frontline aspiration thrombectomy can also benefit significantly from direct routing to endovascular-capable centers. Further work should focus on in-field patient screening tools and controlled trials, e.g., RACECAT (NCT02795962), to provide crucial data to inform pre-hospital management systems for AIS-LVO. In the RACECAT trial, no differences were found between direct vs. transfer patient cohorts in Catalonia admitted for thrombectomy within 7 h of symptom onset (8). While RACECAT provides important data on the efficiency of stroke care in Catalonia, outcomes from the region's single, integrated, public-use healthcare network (which covers the entire Catalonian population of 7.5 million) (21) might not be generalizable to health systems elsewhere in the world, and raises vital questions regarding whether aspects of this system can be adapted to other urban and rural catchments. For instance, the onset-to-puncture times in RACECAT were only delayed by 56 min for transfer patients (onset-to-puncture for transfer was 270 vs. 214 min for direct) (8). By comparison, in our registry, onset-to-puncture time delay was 148 min (339 min for transfer vs. 191 min for direct, *p* < 0.0001) – nearly triple that of RACECAT. This much longer delay in onset-to-puncture time encountered in our registry may explain why in RACECAT, direct and transfer had equivalent outcomes, but in our registry, direct admission patients had significantly higher rates of good functional outcome (mRS 0–2) at 90 days. This point is supported by our multivariable logistic regression (Table 4) which found longer onset-to-puncture (per 30 min increase) to be a significant independent predictor of worse functional outcome at 90 days [OR 0.85 (95% CI 0.74, 0.97), *p* = 0.0180], whereas direct admission was not found to be a significant independent predictor [OR 2.17 (95% CI 0.42, 11.3), *p* = 0.3584].

An important consideration in design of COMPLETE compared to studies such as RACECAT, STRATIS, and DEFUSE-3, is that COMPLETE was a highly inclusive, real-world evaluation of global AIS-LVO treatment. COMPLETE was a global registry that included patients for aspiration thrombectomy with no restrictions on stroke location (anterior/posterior) or onset time. COMPLETE provides real-world data on outcomes based on local systems of care, transfer patterns, and time delays across broad geographies. This experience showed that under current systems of care, direct admission is appropriate for a majority of admitted strokes. However, each locality should develop their own stroke protocols based on the location of, and transit time required to reach a comprehensive stroke center or a stroke center with thrombectomy capability.

Additional research should focus on AIS-LVO detection to facilitate direct routing of patients to appropriate treatment centers. We concur with the need for well-designed, randomized-controlled trials to evaluate AIS-LVO systems of care based on regional access characteristics and patient populations (20, 22).

### Limitations

A limitation of this non-randomized study is the lack of a comparator arm. This study also did not track the decision making process for determining if a patient should be transferred or directly admitted, the distance from location of symptom onset to thrombectomy center (hub), any time metrics related to the initial institution (spoke) for transfer patients (e.g., onset to spoke arrival time, spoke door in to door out time, spoke departure to hub arrival time), nor time of IV-tPA administration, thus limiting possible analyses. Additionally, the conclusions of this analysis may not be widely applicable where primary and comprehensive stroke centers are not similarly accessible for all patients and direct admission may not be an option for many geographical areas.

Nevertheless, this large, prospective registry was core-lab adjudicated, and safety results were assessed by independent medical reviewers. This registry also enrolled patients from a large, heterogeneous population that included posterior lesions, and did not exclude patients based on presentation window.

## Conclusion

In the COMPLETE registry, patients directly admitted to an endovascular-capable center for frontline aspiration thrombectomy experienced significantly faster onset-to-puncture times and better rates of good functional outcome at 90 days.

## Data availability statement

Further inquiries for data can be directed to the corresponding author/s.

## Ethics statement

Informed consent was obtained for all enrolled patients. The study was approved by the appropriate IRB/EC for each participating center. A complete list of all IRB/EC is provided as follows: Institutional Review Board/Ethics Committees The study was approved by the following Institutional Review Boards and Ethics Committees: Comité de protection des personnes Sud-Ouest et Outre-Mer 4, Friedrich-Alexander-Universität Erlangen-Nürnberg, Ethikkommission der Universität zu Lübeck, Ethikkommission bei der Sächsischen Landesärztekammer, Medizinische Fakultät/Universitätsklinikum Magdeburg A. ö. R., Komisja Bioetyczna przy Uniwersytecie Medycznym w Lublinie, Independet Ethics Committee at SBHI [State Budgetary Hospital] n.a. I. V. Davydovsky MHD [Moscow Health Department], Local Ethics Committee of SPb GBUZ City Multidisciplinary Hospital No. 2, Local Ethics Committee at the City Clinical Hospital No. 1 named after N.I. Pirogov No. 9, Comité Ético de Investigación Clínica (CEIC) del Hospital Universitario Vall d'Hebron, CEIC del Hospital Virgen de la Arrixaca, Western Institutional Review Board, University of Miami Human Subject Research Office, Cedars-Sinai, Office of Research Compliance and Quality Improvement, UTHSC (University of Tennessee Health Science Center) IRB, KUMC (University of Kansas Medical Center) Human Research Protection Program IRB, HCA – HealthONE IRB, AdventHealth Orlando IRB, Prisma Health-Midlands IRB, Sutter Health IRB, St Joseph Health IRB, Human Research Committee Partners HealthCare Systems Inc., Dignity Health, NYU School of Medicine's Office of Science and Research Institutional Review Board, MetroWest Medical Center IRB, Ochsner Clinic Foundation IRB, Houston Methodist Research Institute, Naples Community Hospital IRB, SSMSTL (SSM Health Care St Louis) IRB, UT (University of Tennessee)-College of Medicine IRB, Covenant Health IRB, Mercy Health North LLC Adult IRB Research Oversight and Education. The patients/participants provided their written informed consent to participate in this study.

## Author contributions

All authors listed have made a substantial, direct, and intellectual contribution to the work and approved it for publication.

## Funding

This study was funded by Penumbra, Inc (Alameda CA). OZ, JF, and AH were the study's principle investigators and were involved in study design. The sponsor was responsible for database setup, site monitoring, data management, and statistical analysis.

## Conflict of interest

AH: Consultant/Speakers bureau: GE Healthcare, Genentech, Medtronic, Microvention, Penumbra, Stryker, Cerenovus, Viz.ai, Balt and Scientia. OZ: Grant/research support from Genentech, Medtronic Neurovascular, Stryker. Consultant for Codman, Medtronic Neurovascular, National Institutes of Health StrokeNet, Penumbra, Stryker. Honoraria from Codman, Medtronic Neurovascular, Penumbra, Stryker. Serves as an expert witness. Ownership interest in Galaxy Therapeutics, Inc. JF: Grant/research support: Microvention, Penumbra, Stryker. Consultant: Microvention, Stryker. Other financial or material support: Ownership interest: Imperative Care. AN, BA, and AD: None. KW: Consulting and fees from Covidien. AT: Proctor and consultant for Medtronic, Stryker, MicroVention, and Perflow.

## Publisher's note

All claims expressed in this article are solely those of the authors and do not necessarily represent those of their affiliated organizations, or those of the publisher, the editors and the reviewers. Any product that may be evaluated in this article, or claim that may be made by its manufacturer, is not guaranteed or endorsed by the publisher.

## Abbreviations

AIS: acute ischemic stroke
ASPECTS: Alberta stroke program early CT score
ECASS: European cooperative acute stroke study
ENT: embolization in previously uninvolved or new territories
IV: intravenous
LVO: large vessel occlusion
mRS: modified Rankin Score
mTICI: modified thrombolysis in cerebral infarction
MT: mechanical thrombectomy
NIHSS: National Institutes of Health stroke scale
SAE: serious adverse event
sICH: symptomatic intracranial hemorrhage
tPA: tissue plasminogen activator.
